# LncRNA *CDKN2B-AS1*/miR-141/cyclin D network regulates tumor progression and metastasis of renal cell carcinoma

**DOI:** 10.1038/s41419-020-02877-0

**Published:** 2020-08-19

**Authors:** Pritha Dasgupta, Priyanka Kulkarni, Shahana Majid, Yutaka Hashimoto, Marisa Shiina, Varahram Shahryari, Nadeem S. Bhat, Laura Tabatabai, Soichiro Yamamura, Sharanjot Saini, Yuichiro Tanaka, Rajvir Dahiya

**Affiliations:** 1 Department of Urology, Veterans Affairs Medical Center, San Francisco and University of California San Francisco, San Francisco, CA USA; 2 Department of Surgery, University of Miami Miller School of Medicine, Miami, FL USA; 3 Medical College of Georgia, Augusta University, Augusta, GA USA

**Keywords:** Metastasis, Urological cancer

## Abstract

The molecular heterogeneity of renal cell carcinoma (RCC) complicates the therapeutic interventions for advanced metastatic disease and thus its management remains a significant challenge. This study investigates the role of the lncRNA *CDKN2B-AS1* and miR-141-3p interactions in the progression and metastasis of kidney cancer. Human renal cancer cell lines (ACHN and Caki1), normal RPTEC cells, tissue cohorts, and a series of in vitro assays and in vivo mouse model were used for this study. An overexpression of *CDKN2B-AS1* was observed in RCC compared to normal samples in TCGA and our in-house SFVAMC tissue cohorts. Reciprocally, we observed reduced expression of miR-141 in RCC compared to normal in the same cohorts. *CDKN2B-AS1* shares regulatory miR-141 binding sites with *CCND1* and *CCND2* genes. Direct interactions of *CDKN2B-AS1*/miR-141/Cyclin D1–D2 were confirmed by RNA immunoprecipitation and luciferase reporter assays indicating that *CDKN2B-AS1*/miR-141/Cyclin D1–D2 acts as a ceRNA network in RCC. Functionally, attenuation of *CDKN2B-AS1* and/or overexpression of miR-141 inhibited proliferation, clonogenicity, migration/invasion, induced apoptosis in vitro and suppressed tumor growth in xenograft mouse model. Further, overexpression of *CDKN2B-AS1* is positively correlated with poor overall survival of RCC patients. Expression of miR-141 also robustly discriminated malignant from non-malignant tissues and its inhibition in normal RPTEC cells induced pro-cancerous characteristics. *CDKN2B-AS1* attenuation or miR-141 overexpression decreased *CCND1/CCND2* expression, resulting in reduced *RAC1/pPXN* that are involved in migration, invasion and epithelial–mesenchymal transition. This study, for the first time, deciphered the role of *CDKN2B-AS1*/miR-141/Cyclin D axis in RCC and highlights this network as a promising therapeutic target for the regulation of EMT driven metastasis in RCC.

## Introduction

Renal cell carcinoma (RCC) is one of the most common cancers in the USA accounting for nearly 14,830 deaths and 73,750 new cases in 2020^1^. Surgery is the first line of treatment resulting in successful resection and long-term disease-free status with an overall survival rate of more than 60%. However, in approximately 30% of localized RCC cases recurrence occurs with distant metastasis^2^. The obstinate nature of RCC to current treatment regimens is a primary cause of poor prognosis in patients with metastatic recurrence. Lack of sensitivity to both chemotherapy and immunotherapy makes therapeutic options difficult^3–5^. It is, therefore, of utmost importance to improve our understanding of RCC pathogenesis by identifying new biomarkers that lead to better prediction and therapeutic intervention of aggressive RCC^6^.

Emerging lines of evidence suggest that cancer aggressiveness is associated with epithelial–mesenchymal transition (EMT)^7^. It is a well-orchestrated process involved in tumor invasion and metastasis comprising characteristic phenotypic changes through transition from polarized immotile epithelial cells to motile mesenchymal cells^8^. EMT changes in cellular morphology and migratory properties are governed by numerous factors^9^. Increase in mesenchymal properties accompanied by augmented expression of mesenchymal markers like N-cadherin, fibronectin, vimentin and matrix metalloproteinase (MMPs) and decreased expression of epithelial markers, like E-cadherin, α-E-Catenin, claudin etc.^10–13^ are common EMT phenomena. Often the progression of cancer through EMT is significantly induced by the interaction of Cyclin-D with its binding partner, Cdk4 which act as transcriptional regulators controlling cell proliferation and migration^14–16^. It is well known that Cyclin-D regulates the rate-limiting step in cell cycle progression from G1 to S phase. Accumulating evidence also suggest that, abnormal Cyclin-D-Cdk4 over-expression promotes tumor growth and metastasis^17^, but how this correlates with tumor metastasis or controls cell adherence and invasion is poorly understood.

Reports show that non-coding RNAs are involved in the regulation of factors involved in EMT^18^. MicroRNAs (miRNAs), a naturally occurring class of small noncoding RNA molecules of 18-22 nucleotides long^19^, are known to regulate gene expression via both translational inhibition and mRNA degradation^20^ whereas, long noncoding RNAs (lncRNAs), with more than 200 nucleotides, can also act as regulators for tumor-suppressive miRNAs in different cancers^21–23^. Recently, a class of lncRNAs have been categorized as competing endogenous RNA (ceRNA) which involves crosstalk among lncRNAs, mRNAs, and their shared miRNAs. Thus a novel regulatory mechanism is hypothesized suggesting that lncRNAs and mRNAs communicate with each other by competing for common miRNA response elements^24–26^.

In this context, we describe the novel role of lncRNA *CDKN2B-AS1* and miR-141-3p (miR-141) in the regulation of Cyclin-D to govern the metastatic progression of RCC. To our knowledge, this is the first report to directly demonstrate that *CDKN2B-AS1*/miR-141 interaction is a crucial component in RCC progression and metastasis through the Cyclin-D/Rac /paxillin pathway.

## Materials and methods

### Cell lines and cell culture

The normal RPTEC (ATCC number: CRL-4031) and renal cancer ACHN (ATCC number: CRL-1611) and Caki1 (ATCC number HTB-46) cell lines were purchased from the ATCC (Manassas, VA). These human-derived cell lines were authenticated by DNA short-tandem repeat analysis by ATCC. Cell line experiments were performed within 5–6 months of their procurement/resuscitation. ACHN cells were cultured in MEM media, Caki1 cells in and McCoy 5A medium, and RPTEC cells in DMEM:F12 Medium (ATCC^®^ 30-2006™). All media were supplemented with 10% FBS and 1X antibiotics (penicillin and streptomycin). Cell lines were maintained at 37 °C and humidified atmosphere of 5% CO_2_.

### miRNA/siRNA transfections

To induce overexpression or knockdown, cells were transiently transfected with either mirVana miRNA Mimic (50 nmol/L), or anti-miR miRNA inhibitor (50 nmol/L) (Thermo Fisher Scientific), and 30 nmol/L of siRNA (Sigma Aldrich) using Lipofectamine RNAi Max (Thermo Fisher Scientific) according to the manufacturers’ protocol. To verify transfection efficiency, mirVana miRNA Mimic Negative Control #1, miRNA inhibitor control and siRNA control were used respectively in each transfection experiment at the same concentration. All transfection experiments were carried out for 72 h.

### Clinical specimens

Formaldehyde-fixed-paraffin-embedded (FFPE) tissue specimens from patients undergoing radical nephrectomy were obtained from the San Francisco Veterans Affairs Medical Center (SFVAMC). Written informed consent was obtained from all patients and the study was in accordance with institutional guidelines (IRB approval no: 16-18555). All patient samples were pathologically confirmed for clear cell RCC (cc-RCC), and slides were reviewed by a board-certified pathologist for the identification of tumor foci and adjacent normal tissue. Apart from SFVAMC cohort, TCGA-KIRC, TCGA-KICH, TCGA-KIRP, ICGC, and GEO cohorts for RCC (from online databases) were also used to check the expression levels.

### RNA/miRNA extraction and quantitative real-time PCR (qRT-PCR)

Total RNA was extracted from microdissected FFPE tissues and cell lines using miRNeasy FFPE and miRNeasy mini kits (Qiagen) respectively in accordance to manufacturer’s instructions. Mature miRNA and mRNAs were assayed by qRT-PCR using QuantStudio 7 Flex-Real Time PCR System (Applied Biosystem) using Fast SYBR® Green Master Mix/TaqMan universal PCR master mix, probes and primers (Applied Biosystems Inc., Foster City, CA, USA) following manufacturer’s protocol. Human GAPDH and RNU48 were used as endogenous controls, and relative expression of RNA/miRNA were calculated using comparative Ct (threshold cycle). Primer sequences are provided in Supplementary Table T1.

### DNA methylation analysis in-silico, in cell lines, and 5Aza-CdR treatment

DNA hypermethylation of the miR-141 promoter in normal and RCC samples was first confirmed in the TCGA data-base using Wanderer software^27^. In order to confirm the methylation status of the miR-141 promoter in RCC cell lines, we extracted DNA from ACHN and Caki1 using DNeasy tissue kit (Qiagen). Sodium bisulphite modification was done using EZ DNA methylation-Gold kit (Zymo Research, Orange, CA, USA) following the manufacturer’s protocol. Bisulfite-treated DNA was analyzed by methylation-specific quantitative polymerase chain reaction (MS-qPCR) with primer pairs specific for methylated and unmethylated regions of the miR141 promoter. MS-qPCR was performed as described earlier^28^. For each sample, the percent of methylation was calculated by the difference of Ct in methylated sample (Ct-M) and Ct in unmethylated sample (Ct-U). The primers sequences are mentioned in Supplementary Table 1. ACHN and Caki1 cells were treated daily with 20 μmol/L 5-AZA-deoxycytidine (5Aza-CdR) (Sigma-Aldrich) for 72 h^29^ and total RNA was isolated using a miRNeasy mini kit (Qiagen) to check miR-141 expression.

### Cell viability, clonability, migratory, invasion, and apoptosis assays

Cell viability was measured at 24, 48, and 72 h using a CellTiter 96 Aqueous Solution Cell Proliferation Assay kit (Promega, Madison, WI) following the manufacturer’s instructions. For colony formation assay, cells were seeded at a low density (1000 cells/plate) after 72 h of transfection and were allowed to grow until visible colonies were formed. Plates were then stained with giemsa followed by crystal violet and colonies were counted. Culture inserts of 8-µm pore size (Transwell; Costar) were used for migration and invasion assay. Inserts were coated with Matrigel (BD Biosciences) (100 µg/well) for invasion. Briefly, 72 h post-transfection, cells were counted and placed on inserts at 0.5 × 10^5^ cells/ml (for migration) and 1 × 10^5^ (for invasion) in serum-free medium and were allowed to migrate/invade for 48–72 h at 37 °C. Cells migrated or invaded through the pores were fixed, stained with 0.05% crystal violet. Crystal violet was solubilized with methanol, and quantified at 540 nm by a kinetic microplate reader (Spectra MAX 190; Molecular Devices). FACS analysis for apoptosis was done 72 h post-transfection, using Annexin V-FITC and 7-AAD Kit (Beckman Coulter, Inc.) in accordance with the manufacturer’s instructions. Cold PBS washed cells were resuspended in 1X binding buffer and stained with Annexin V-FITC/7AAD viability dye. After 30 min of incubation at room temperature in the dark, stained cells were analyzed using BD FACSVerse (BD Pharmingen).

### Dual-luciferase reporter assay

The wild type (WT) and off-target (OT) luciferase reporter constructs were made by ligating annealed custom oligonucleotides containing putative target binding sites and corresponding nontarget /mutant sites into the pmiR-GLO reporter vector. Luciferase constructs (0.1 µg) were cotransfected into ACHN and Caki1 cells along with 50 nmol/L miR-141 mimic or control-miR using transfection reagent JetPrime (Polyplus-transfection, Illkirch, France). Luciferase activities were measured using the Dual-luciferase assay (Promega, Madison, WI) 48 h post transfection. Relative luciferase activity was calculated by normalizing Firefly luciferase to Renilla luminescence.

### RNA immunoprecipitation (RIP) assay

Simultaneous binding of miR-141 to lncRNA and mRNA was confirmed by RIP assay. An imprint RIP kit was used following the manufacturer’s protocol (Sigma-Aldrich, St. Louis, MO, USA). IgG (control) and Ago2 antibodies were used for immunoprecipitation. The immunoprecipitated RNA fraction was reverse transcribed to cDNA using High capacity cDNA reverse transcription kit (Thermo Fisher). Fold enrichment of lncRNA and mRNA to Ago2 with respect to IgG was calculated using quantitative RT-PCR.

### Western blot and immunofluorescence analysis

Total protein extraction was performed as described previously^18^. Proteins were then separated by NuPAGE 4–12% Bis-Tris Protein Gels (Invitrogen) and subsequently transferred onto nitrocellulose membrane. Resulting blots were blocked using Odyssey blocking buffer (LI-COR) and subsequently probed with primary and secondary antibodies. Blots were scanned using an Odyssey Infrared Imaging System Scan and quantification was carried out with the LI-COR Odyssey® scanner and software (LI-COR Biosciences). The primary antibodies used are listed in Supplementary Table 2. For immunofluorescence, transiently transfected ACHN and Caki1 cells were fixed in 4% paraformaldehyde for 15 min followed by blocking (1X PBS/5% normal goat serum/0.3% Triton X-100) for 1 h at room temperature. Cells were then incubated overnight in 1:100 fold diluted primary antibody at 4 °C. Cells were then reprobed with 1:300 fold diluted secondary antibody for 2 h and counter stained with 0.5 µg/ml of 4′,6-diamidino-2-phenylindole (DAPI) for 5 min. Cells were then mounted on a slide using prolong gold anti-fade reagent. Images were captured using Zeiss microscope (model: Axio Imager.D2).

### Renal cancer xenografts

We studied the anti-tumorigenic effects of miR-141 in established tumors using a renal cancer xenograft nude mouse model as previously described^6,30^. Male nude mice (4–5 week-old, *n* = 10; Charles River Lab) were subcutaneously injected with 1 × 10^7^ Caki1 cells. Once palpable tumors were formed, mice were randomized in two groups for the treatment and control groups (five in each). Synthetic miRNA (miR-141 mimic/miR-CON) of 6.25 μg was complexed with 1.6 μL siPORTamine transfection reagent (Ambion) in 50 μL PBS, and delivered intratumorally in 3-day intervals. Tumor volume was calculated according to the formula {(*x*)^2^ * *y*}/2 where *x* < *y* (*x* = width; *y* = length). Experiments were terminated 3 days after the last treatment (day 38). Tumor measurements and statistical analysis were performed by researchers who were blinded for the control and treatment groups. All animal care was in accordance with institutional guidelines (IACUC approval no: 16-004).

### Statistical analysis

All quantified data represents an average of at least three independent experiments or as indicated. Statistical analyses were performed using GraphPad Prism 7 and MedCalc. Error bars represent ± standard error mean (S.E.M). The Mann–Whitney *U* test was used to assess the difference between miRNA expressions in tumor/normal adjacent tissue. Significant differences between the groups were determined using the Student’s *t*-test. All tests were performed either one tailed or two tailed and results were considered statistically significant at *P* ≤ 0.05. Receiver operating curves (ROC) were performed to evaluate the potential of miR-141 to differentiate between malignant and non-malignant samples using MedCalc software showing area under curve (AUC) and 95% confidence interval. Kaplan–Meier analyses for overall survival with respect to miR-141 methylation levels in TCGA-KIRC cohort were generated using software “EZR” (10.1038/bmt.2012.244). Tumor measurements and statistical analyses for all experiments were performed blindly for the control and treatment groups.

## Results

### lncRNA *CDKN2B-AS1* is oncogenic and is a direct target of miR-141

**Fig. 1:**
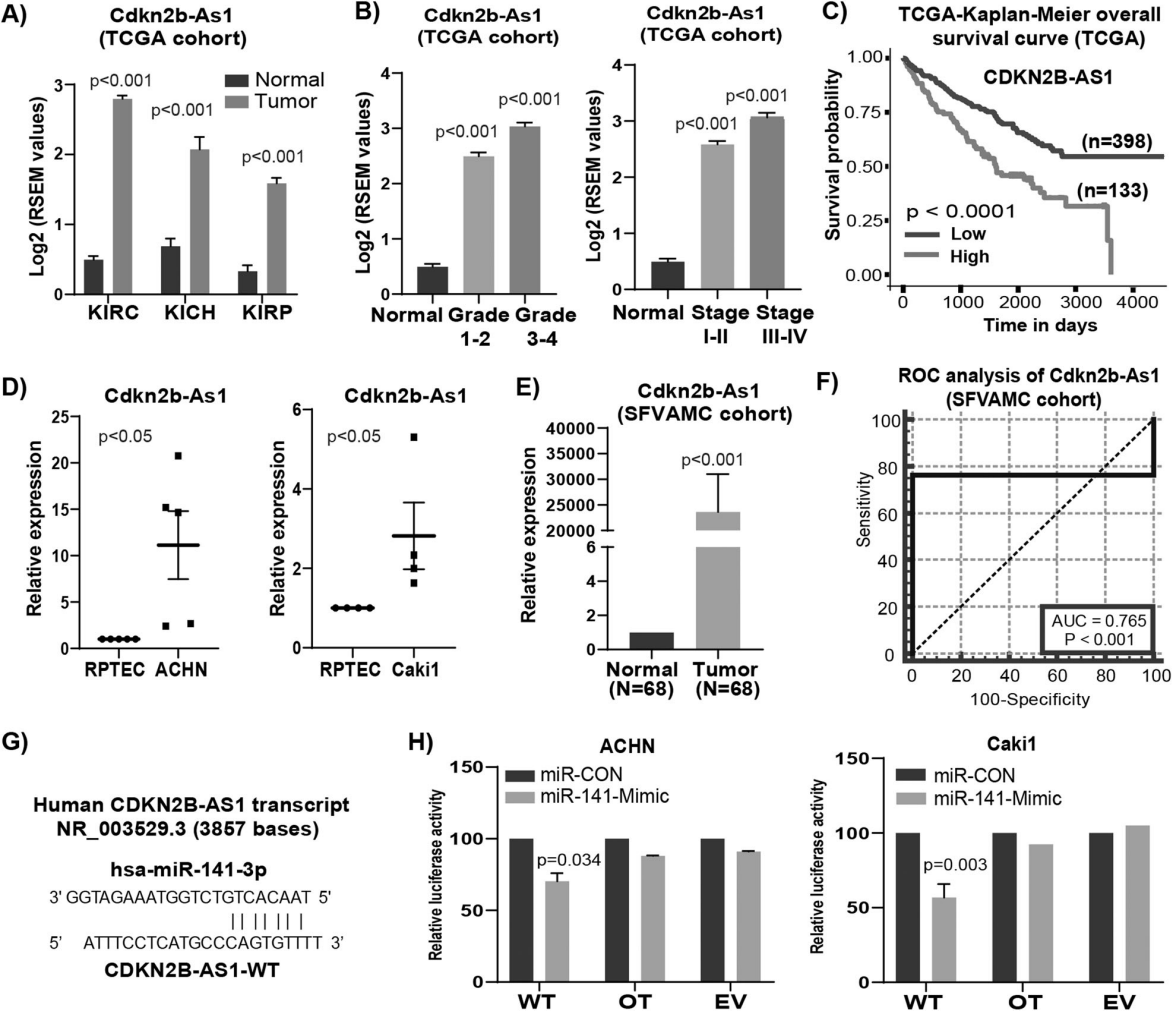
lncRNA *CDKN2B-AS1* is oncogenic in renal cancer and is a direct target of miR-141. **a** Expression levels of *CDKN2B-AS1* among KIRC (normal = 72, tumor = 518), KICH (normal = 25, tumor = 66) and KIRP (normal = 30, tumor = 197) patient samples in TCGA cohort using Wanderer software (*P*-value calculated by Mann–Whitney two-tailed test). **b** Expression of *CDKN2B-AS1* in TCGA-KIRC cohort among different grades (normal = 72, grade 1–2 = 214, grade 3–4 = 268) and stages (normal = 72, stage I–II = 288 and stage III–IV = 203). **c** Overall survival in TCGA-KIRC cohort as performed by Kaplan–Meier analysis (using UALCAN software). **d** Relative expression levels of lncRNA *CDKN2B-AS1* in RCC cell lines ACHN and Caki1. **e** Expression *CDKN2B-AS1* in matched pairs of RCC tissue samples from SFVAMC cohort (P-value calculated by Mann–Whitney two-tailed test). **f** Receiver operating curve (ROC) analysis on SFVAMC cohort showing ability of lncRNA *CDKN2B-AS1* to differentiate between malignant and non-malignant samples (SFVAMC cohort). **g** Predicted binding sites of miR-141 in *CDKN2B-AS1* sequence. **h** Luciferase assays showing decreased reporter activity after co-transfection with either wild-type (WT), off-target (OT) *CDKN2B-AS1* or luciferase control constructs (EV) with miR-CON/miR-141 in ACHN and Caki1 cells.

Initially, we found *CDKN2B-AS1* is an oncogenic lncRNA in RCC based on TCGA (Fig. 1a), ICGC and GEO databases (Fig. S1). TCGA cohort also revealed that high *CDKN2B-AS1* expression increases from lower grade and stage to higher grade and stage (Fig. 1b). Moreover, higher expression is significantly (*p* < 0.0001) correlated to overall survival (Fig. 1c). In agreement with these data cohorts, significantly higher *CDKN2B-AS1* expression was also seen in RCC cell lines ACHN, Caki-1 as compared to normal RPTEC (Fig. 1d) and SFVAMC cohort (Fig. 1e). Patient and tumor characteristics are summarized in Supplementary Table T3. ROC analysis shows an AUC of 0.765 (*P* < 0.001; 95% CI = 0.684–0.833) (Fig. 1f) suggesting the diagnostic potential of *CDKN2B-AS1* to discriminate between normal and tumor tissues. We used computational algorithms and identified putative miR-141 binding sites in the *CDKN2B-AS1* sequence (Fig. 1g). To examine potential miR-141/*CDKN2B-AS1* interaction experimentally, we performed luciferase reporter assay. Both ACHN and Caki1 cells co-transfected with miR-141 and *CDKN2B-AS1* wild type binding site revealed a consistent reduction of luciferase activity suggesting that miR-141 directly interacts and regulates *CDKN2B-AS1* (Fig. 1h). Thus, all these data suggest that clinically important *CDKN2B-AS1* is an oncogenic lncRNA in RCC and is a novel target of miR-141.

### *CDKN2B-AS1* inhibition by siRNA suppresses tumorigenicity in RCC

**Fig. 2:**
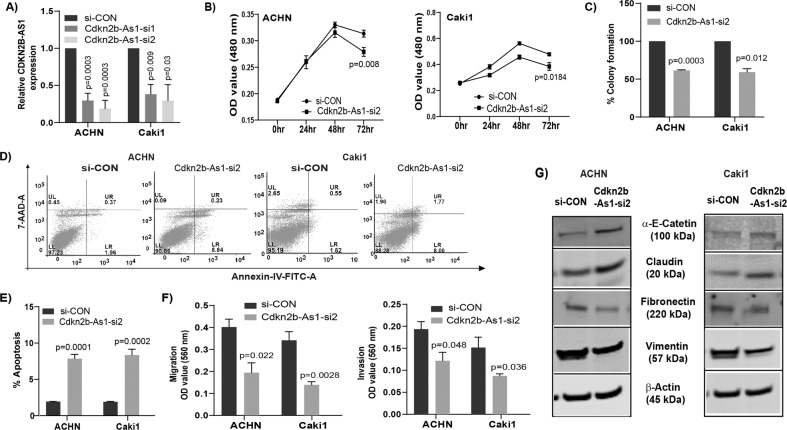
Knockdown of lncRNA *CDKN2B-AS1* reduces tumorigenicity in renal cancer. **a** Relative expression of *CDKN2B-AS1* in ACHN and Caki1 cells transfected with *CDKN2B-AS1* siRNAs. **b** Cell proliferation assessed by MTS assay after knockdown of *CDKN2B-AS1* in both cell lines with siRNA-2. (C) Graphical representation showing knockdown of *CDKN2B-AS1* with siRNA-2 significantly decreased colony formation in ACHN and Caki1 cells. **d**, **e** ACHN and Caki1 cell lines showing significant induction of apoptosis (early + late) compared to control after knockdown of *CDKN2B-AS1*. **f** Reduced migration and invasion in *CDKN2B-AS1* siRNA transfected cells compared to control treatment. **g** Changes in EMT related proteins in both ACHN and Caki1after knockdown of *CDKN2B-AS1*.

Transient transfection of ACHN and Caki1 cells with *CDKN2B-AS1* siRNAs for 72 h showed significant reduction in *CDKN2B-AS1* expression (Fig. 2a). *CDKN2B-AS1* knockdown in both cell lines significantly inhibited cell proliferation (Figs. 2b, S2A), and clonogenic survival (Figs. 2c, S2B) with a significant increase in apoptosis (Fig. 2d, e). Decreased cell migration/invasion (Figs. 2f, S2C, D) with simultaneous changes in EMT markers such as an increase in epithelial markers (α-E-Catenin and claudin) and decrease in mesenchymal markers (vimentin and fibronectin) were also observed (Figs. 2g, S3).

### Expression of miR-141 and its clinical importance in renal carcinoma

**Fig. 3:**
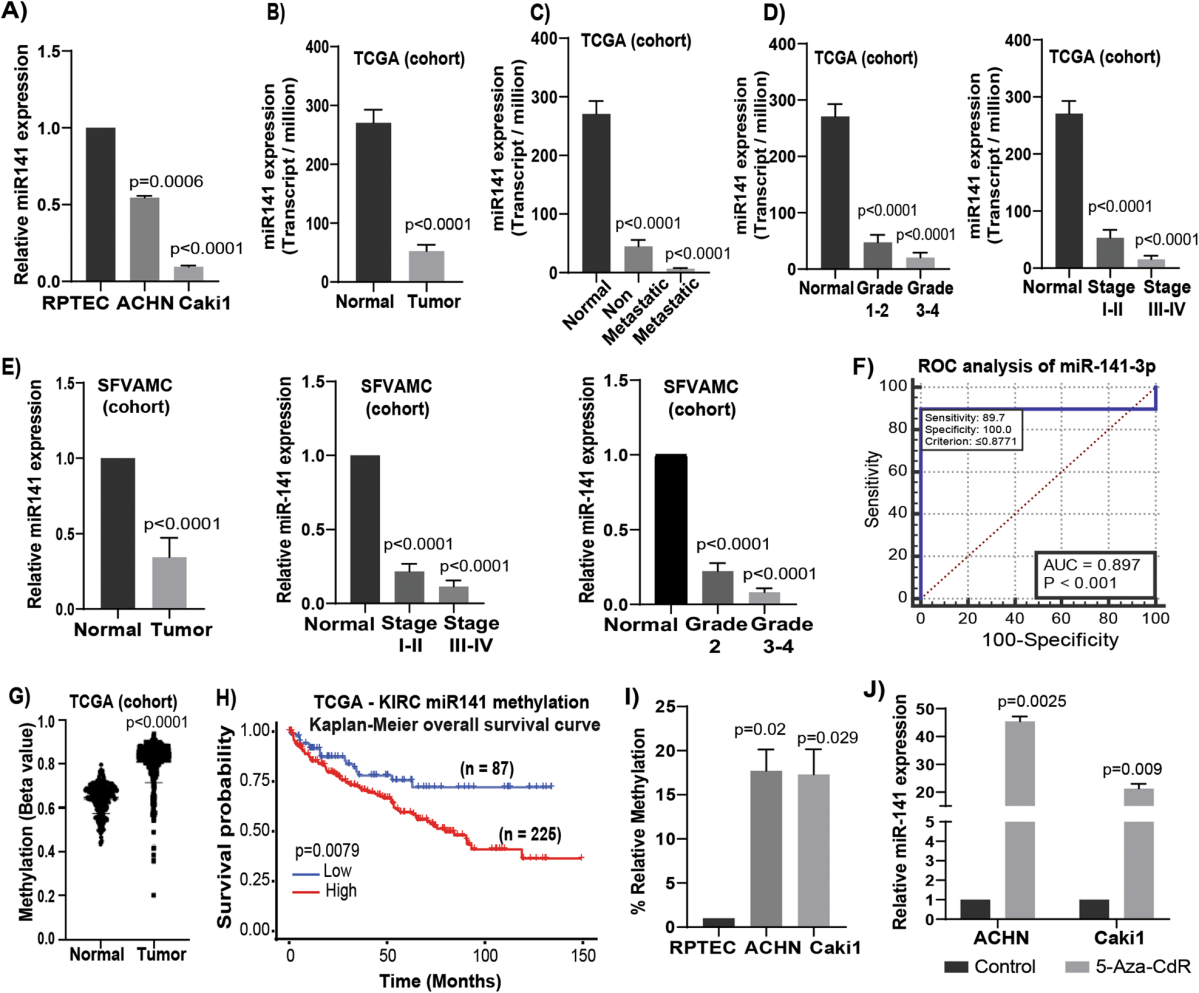
Expression, clinical significance, and epigenetic regulation of miR-141 in renal cancer. **a** miR-141 expression levels in ACHN, Caki1 and RPTEC cells. **b** Expression levels of miR-141 in KIRC-TCGA cohort (normal = 71 and tumor = 544). **c** Expression levels of miR-141 in normal (*n* = 71), non-metastatic (*n* = 405) metastatic (*n* = 77) KIRC patient samples in TCGA cohort. **d** Expression levels of miR-141 in KIRC-TCGA cohort among different grades (normal = 71, grade 1–2 = 297, grade 3–4 = 272) and stages (normal = 71, stage I–II = 296 and stage III–IV = 204). **e** Relative miR-141 expression in RCC tissue *vs* matched adjacent normal regions (*n* = 68), among different grades (normal = 68, grade 2 = 44, grade 3–4 = 22) and in different stages (normal = 68, stage I–II = 52, stage III–IV = 12) as assessed by qRT-PCR (SFVAMC cohort). **f** Receiver operating curve (ROC) analysis showing ability of miR-141 to differentiate between malignant and non-malignant samples. **g** Methylation for KIRC patient samples in TCGA-KIRC cohort for probe cg02624246. **h** Overall survival with TCGA-KIRC methylation as performed by Kaplan–Meier analysis. **i** Methylation status of miR-141 promoter in RCC cell lines compared to non-malignant RPTEC as assessed by MS-qPCR. **j** Expression of miR-141 in 5-Aza-CdR treated and untreated ACHN and Caki1 cell lines. Results are representative of three independent experiments. *P* value calculated by Student *t* test. Bar = mean ± standard error mean (SEM).

Since our results confirmed *CDKN2B-AS1* as a direct target of miR-141, we examined miR-141 status and clinical importance in RCC. Expression of miR-141 was significantly down-regulated in RCC cell lines (Fig. 3a) and in tumor samples (Fig. 3b–e) compared to normal cell line and samples. Lower expressions significantly decreased with increasing grade, stages and in metastatic compared to non-metastatic tumors (Fig. 3c–e). Patient and tumor characteristics are summarized in Supplementary Table T3. ROC analysis showed an AUC of 0.897 (*P* < 0.001; 95% CI = 0.833–0.943) (Fig. 3f) suggesting that miR-141 can be used as a potential diagnostic parameter to discriminate between normal and tumor tissues.

### Epigenetic regulation of the miR-141 locus

We identified a genomic site rich in CpG island located upstream of the miR-141 in chromosome 12p13. In the TCGA cohort, we observed hypermethylation of miR-141 promoter in tumor tissues as compared to normal (Figs. 3g, S4A) which is significantly associated with poor patient survival (Fig. 3h). Similarly, RCC cell lines ACHN and Caki1 also showed hypermethylation compared to normal RPTEC cells (Fig. 3i). Further, we treated ACHN and Caki1 cell lines with demethylating agent 5-Aza-CdR and observed decrease in methylation (Fig. S4B) with concomitant increase in miR-141 expression (Fig. 3j) indicating possible epigenetic regulation. A significant decrease in the expression of methylation regulatory genes such as DNMTl, DNMT3a, and DNMT3b were also noted after 5-Aza-CdR treatment compared to control (DMSO) in both ACHN and Caki1 cell lines^31^.

### miR-141 over-expression phenocopies functional effects obtained with *CDKN2B-AS1* inhibition in vitro and suppresses tumorigenicity and in vivo

**Fig. 4:**
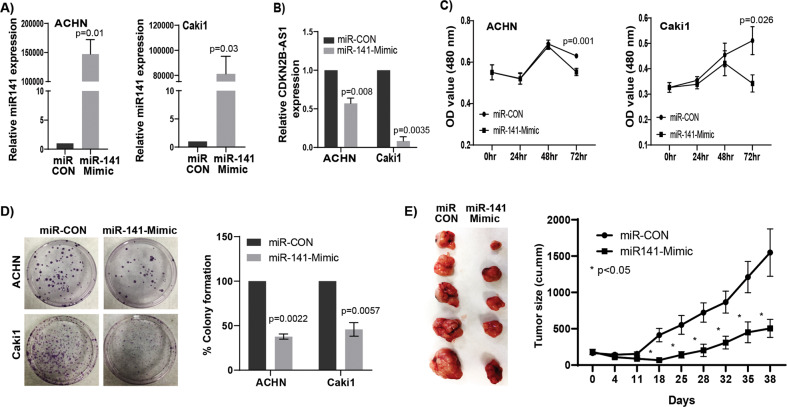
miR-141 over-expression mimics the knockdown effect of lncRNA *CDKN2B-AS1* in vitro and reduces tumorigenicity in vivo. **a** Relative expression of miR-141 in ACHN and Caki1 cells transfected with miR-141 mimic and control. **b** Significant decrease in *CDKN2B-AS1* expression compared to control in both cell lines overexpressed with miR-141. **c** RCC cell proliferation after transfecting with miR-141 mimic and control as assessed by MTS assay. **d** Colony formation and its graphical representation in miR-141 overexpressing ACHN and Caki1 cells compared to controls. **e** Pictures of excised tumors are taken at the termination of experiment (day 38). Graph represents tumor volume after intratumoral injection of control or miR-141 mimic into established tumors. Injection was started at day 0 and was followed for 38 days. Each mouse in both groups (miR-CON and miR141-3p-Mimic) received a total of eleven injections intermittently. Data represent the mean of each group and error bars are SEM. Results are representative of three independent experiments. *P* value calculated by Student *t* test. Bar = mean ± standard error mean (SEM).

**Fig. 5:**
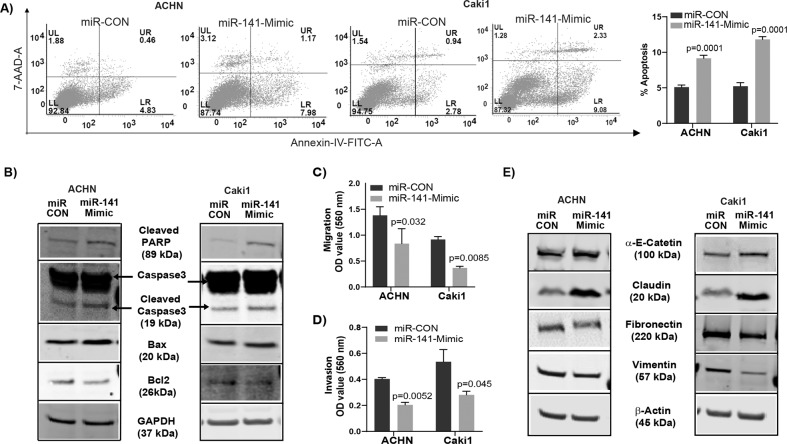
Ectopic miR-141 expression induces apoptosis and inhibits migration/invasion in renal cancer cells. **a** Apoptosis assessed by flow cytometric analysis of annexin-V-FITC -7AAD–stained ACHN and Caki1 cells transfected with miR-CON/miR-141-Mimic. Graph represents total apoptosis (early + late). **b** Immunoblots showing apoptotic proteins in miR-CON/miR-141-Mimic treated ACHN and Caki1 cells with GAPDH as endogenous control. **c** Migration assay and **d** invasion assay as seen in pictures and graphical representation of both ACHN and Caki1 cells after miR-141 overexpression compared to control. **e** Immunoblot assay showing EMT related proteins in miR-CON/miR-141-Mimic treated ACHN and Caki1 cells with β-actin as endogenous control. Graphs are average of three independent experiments. *P* value calculated by Student *t* test. Bar = mean ± standard error mean (SEM).

We sought to determine if *CDKN2B-AS1* causes its anti-tumorigenic effects through miR-141. We checked the effect of miR-141 overexpression in RCC cells. Transient transfection of miR-141 mimic in ACHN and Caki1 cells for 72 h led to over expression of miR-141 compared to control (miR-CON) (Fig. 4a). Also, overexpression of miR-141 significantly reduced *CDKN2B-AS1* expression (Fig. 4b) indicating a reciprocal correlation between miR-141 and *CDKN2B-AS1*. A significant decrease in cell proliferation over time (Fig. 4c) and marked decrease in clonogenicity (Fig. 4d) compared to controls were also observed. Further, we studied the therapeutic potential of miR-141 in a mouse xenograft model. A significant decrease in tumor growth was observed by intratumoral delivery of miR-141 mimic compared to control over the course of experiment. Average tumor volume in the control group was 1548.4 mm^3^ compared to 504.7 mm^3^ in mice that received miR-141 mimic (Fig. 4e). In addition, miR-141 over-expression significantly induced apoptosis with a concomitant decrease in the viable population in both RCC cell lines compared to control (Fig. 5a). This pro-apoptotic role was supported by the induction of cleaved caspase-3, cleaved poly-ADP-ribose polymerase (PARP), an increase in BAX, and a decrease in BCl_2_ at protein levels (Fig. 5b). A significant decrease in migration (Fig. 5c) and invasion (Fig. 5d) was also observed in both RCC cell lines with miR-141 overexpression. We also examined EMT markers as change in migration and invasion are directly associated with EMT. Our results showed an increase in epithelial markers α-E-catenin and claudin with concomitant decrease in mesenchymal markers fibronectin and vimentin at both protein (Fig. 5e) and mRNA (Fig. S5) levels. Taken together, overexpression of miR-141 phenocopies the functional effects of *CDKN2B-AS1* inhibition in vitro and tumor growth suppression effects in vivo.

### miR-141/*CDKN2B-AS1* interaction negatively regulates Cyclin-D and its downstream effectors in RCC

**Fig. 6:**
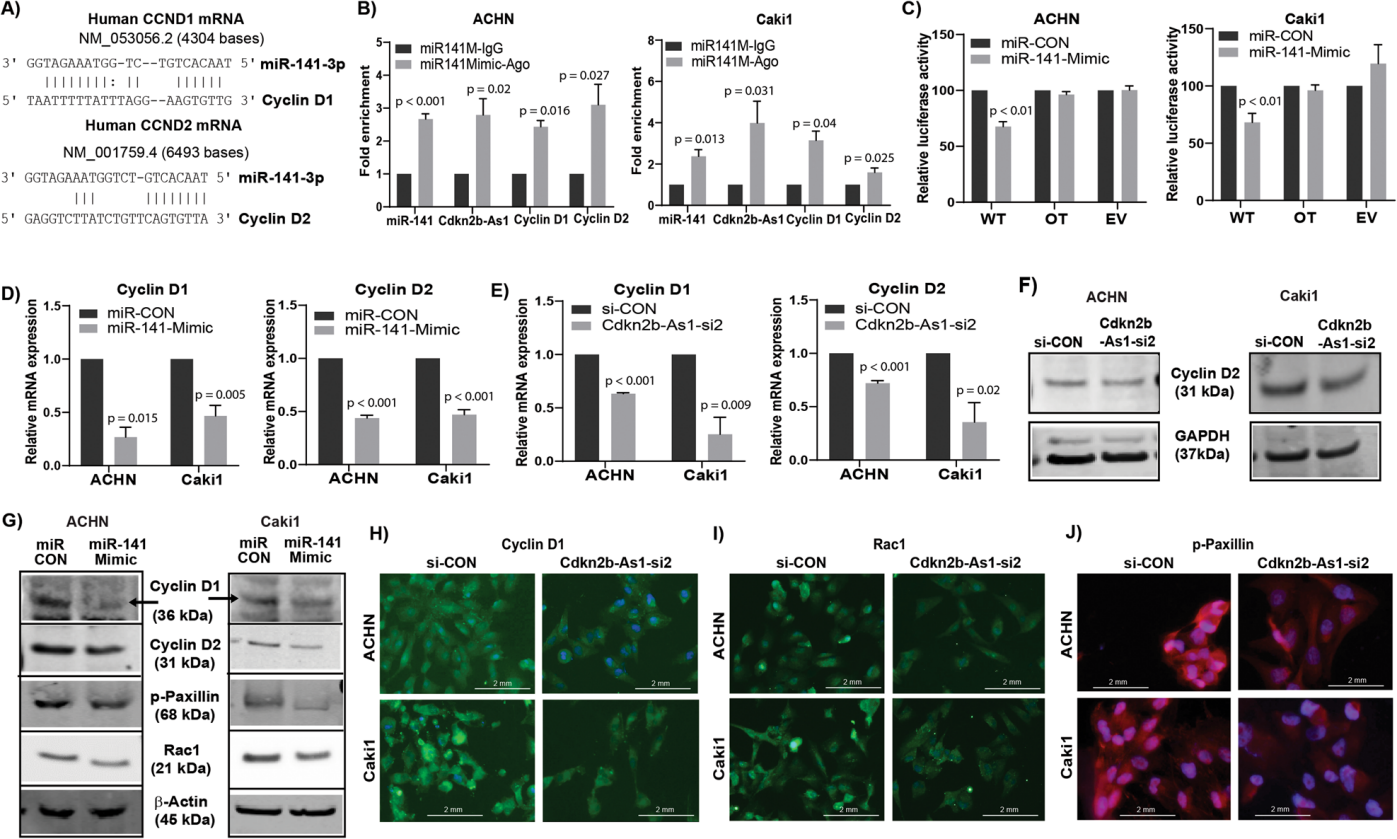
miR-141/*CDKN2B-AS1* interaction negatively regulates Cyclin-D and its downstream effectors in RCC. **a** Predicted binding sites of miR-141 in cyclin-D1 and cyclin-D2 sequences. **b** RIP assay using Ago2 and IgG antibody (control) showing fold enrichment of miR-141, cyclin-D1, cyclin-D2, and CDKN2B-AS1 to Ago2 compared IgG controls in miR-141 overexpressing RCC cells. **c** Luciferase assays showing decreased reporter activity after co-transfection with either wild-type (WT), off-target (OT) cyclin-D2 or luciferase control constructs (EV) with miR-CON/miR-141 in ACHN and Caki1 cells. **d**, **e** Relative mRNA expression of cyclin-D1 and cyclin-D2 in both cell lines after miR-141 overexpression and *CDKN2B-AS1* knockdown respectively. **f** Immunoblots showing changes in cyclin-D2 protein after *CDKN2B-AS1* knockdown in ACHN and Caki1 cells. **g** Western blot analysis showing protein levels of cyclin-D1, cyclin-D2, pPaxillin, rac1, and β-actin (control) in ACHN and Caki1 cells overexpressing miR-141. **h**–**j** Immunostaining of cyclin-D1, rac1 (green) and pPaxillin (red) counterstained with DAPI (blue) in ACHN and Caki1 cells after transfecting with si-CON/CDKN2B-AS1-si2, scale bar: 2 mm (right bottom).

Like *CDKN2B-AS1*, cyclin-D1/cyclin-D2 are also oncogenic in RCC (Fig. S6); and are direct targets of miR-141. As discussed earlier, lncRNAs can act as ceRNAs to carry out their regulatory functions^32–35^. We observed that *CDKN2B-AS1* shared regulatory miR-141 binding sites with cyclin-D1/cyclin-D2 (Fig. 6a), and thereby sponges miR-141 allowing cyclin-D1/D2 to be expressed in tumors. To determine potential miR-141/*CDKN2B-AS1*/Cyclin-D interaction experimentally, we performed RIP assay. Both ACHN and Caki1 cells over expressing miR-141 revealed significant enrichment of cyclin-D1, cyclin-D2, and *CDKN2B-AS1* with Ago2 as compared to IgG control (Fig. 6b). Moreover, decreased luciferase activity also confirmed direct binding of miR-141 to cyclin-D in miR-141 overexpressing ACHN and Caki1 cells compared to controls (Fig. 6c). We also found that overexpression of miR-141 or inhibition of *CDKN2B-AS1* significantly decreased cyclin-D1/D2 expression at both the mRNA (Fig. 6d, e) and protein levels (Figs. 6f–h, S8A, B). This effect was significantly attenuated by miR-141 inhibitor (Fig. S7), indicating that cyclin-D expression is dependent on the interaction between miR-141 and *CDKN2B-AS1*. We further observed a decrease in Rac1, a small GTPase, and a reduction in the phosphorylation of paxillin, a focal adhesion protein, at mRNA levels (Figs. S3, S5) and protein levels (Figs. 6g, I–j, S8C–F) in both miR-141 overexpressed and *CDKN2B-AS1* inhibited RCC cell lines which in turn are involved in regulating cellular migration/invasion. Cumulatively, these results indicate that suppression of *CDKN2B-AS1* by miR-141 inhibits renal cell proliferation, invasion and migration by inhibiting cyclin-D, Rac1, and phosphorylation of paxillin.

### Attenuation of miR-141 exerts tumorigenic attributes in normal RPTEC cells

**Fig. 7:**
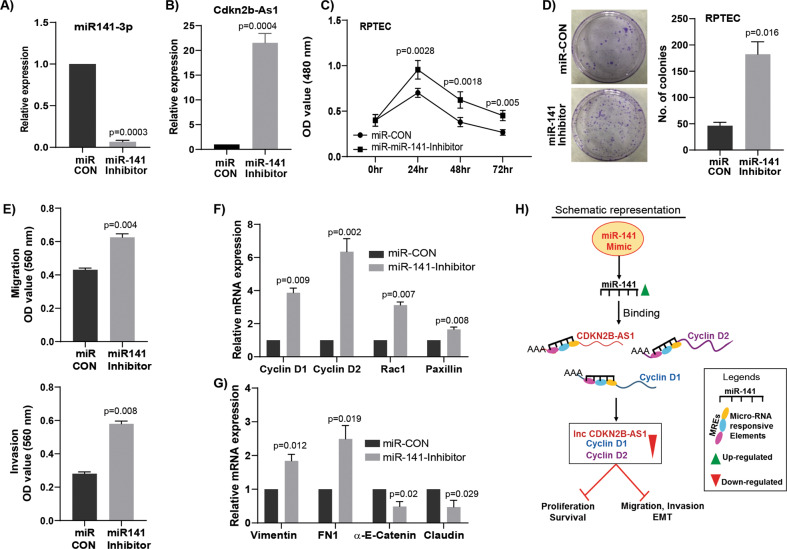
Inhibition of miR-141 exerts tumorigenic attributes in nonmalignant RPTEC cells. **a** Relative miR-141 expression after transient transfection of miR-141 inhibitor compared to miRNA inhibitor control (miR-CON) in non-malignant RPTEC cells. **b** Relative expression of *CDKN2B-AS1* in miR-141 inhibited RPTEC cells. **c** miR-141 inhibition in RPTEC cells induced cell proliferation. **d** Increased colony formation in miR-141 inhibited RPTEC cells as compared to control. **e** An increase in migration and invasion of miR-141 inhibited RPTEC cells. **f**, **g** A significant increase in the mRNA expression of cyclin-D1, cyclin-D2, rac1, paxillin, vimentin, fibronectin (FN1) along with decrease in the expression of α-E-catenin and claudin in RPTEC cells with inhibition of miR-141 compared to control. **h** Schematic representation showing knockdown of *CDKN2B-AS1* due to overexpression of miR-141 results in decreased cyclin-D1 and cyclin-D2 expression which in turn inhibits proliferation/survival/invasion and migration pathways.

We next determined whether attenuation of miR-141 induces tumorigenic characteristics in normal RPTEC cells by targeting *CDKN2B-AS1* and cyclin-D. Transient transfection of miR-141 inhibitor indeed showed a significant decrease in miR-141 expression (Fig. 7a) and an increase in *CDKN2B-AS1* expression (Fig. 7b) along with other pro-cancerous phenotypes such as increased cell proliferation (Fig. 7c), colony formation (Fig. 7d), migration and invasion (Fig. 7e) as compared to controls. Additionally, a significant increase in cyclin-D1, cyclin-D2, rac1, and paxillin (Pxn) expressions were observed in miR-141 inhibited RPTEC cells (Fig. 7f). A noticeable increase in pro-metastatic fibronectin and vimentin with a concomitant decrease in anti-metastatic claudin and α-E-catenin genes were also observed in miR-141 inhibited RPTEC cells compared to controls (Fig. 7g).

## Discussion

Prior studies have shown the regulatory role of noncoding RNAs in tumorigenesis especially in the EMT pathway leading to cancer aggressiveness. *CDKN2B-AS1*, also known as *ANRIL*, is located at chromosome 9p21. *CDKN2B-AS1* is reported to be upregulated in tumor tissues and function as an oncogenic lncRNA in pancreatic, ovarian and laryngeal squamous cell carcinoma^36–38^. Human miR-141 is located at chromosome 12p13.31 and is transcribed from a miR-200 family cluster. Interestingly, expression of miR-141 is controversial since it exhibits either oncogenic^39–41^ or tumor suppressive roles^42–44^ in specific types of cancer. The prime goal of the present study was to understand the role of *CDKN2B-AS1*/miR-141 interactions in regulating RCC progression and metastasis.

In this study, we identify *CDKN2B-AS1* to be a crucial oncogenic lncRNA that plays an important role in renal carcinogenesis. *CDKN2B-AS1* is significantly over-expressed in RCC and the expression increases from lower to higher grades and stages. LncRNA *CDKN2B-AS1* directly interacted with miR-141 as it was found to be a novel target of miR-141. In contrast to *CDKN2B-AS1*, we observed significant attenuation of miR-141 expression in RCC cell lines and tumor samples compared to normal cell line or matched normal samples. As it is known that extensive DNA hypermethylation of CpG islands is highly correlated to activation of cancer-specific genes^45^, we checked the methylation status of miR-141 in normal and RCC tissues. Interestingly, in-silico analysis showed the presence of CpG island in the promoter region of miR-141 and we also found hypermethylation of miR-141 in TCGA samples as compared to normal. This hypermethylation is also found to be significantly associated with poor survival of patients. Similar results were also observed in RCC cell lines compared to a normal RPTEC cell line. Functionally, inhibition of *CDKN2B-AS1* and/or over-expression of miR-141 significantly inhibits the tumorigenic characteristics such as cell proliferation, clonogenicity, migration and invasion whereas induces anticancer apoptotic phenotype in RCC in vitro. In vivo data show suppression of tumor growth by miR-141 overexpression. Conversely, attenuation of miR-141 in normal RPTEC cells induced precancerous characteristics indicated by increased proliferation, migration and invasion.

From a clinical point of view, non-coding RNAs signatures are powerful tools for early cancer diagnosis making them attractive candidates as diagnostic and prognostic biomarkers^18,46,47^. Our results revealed that higher expression of *CDKN2B-AS1* is positively correlated with poor overall survival probability of RCC patients indicating its prognostic capability. *CDKN2B-AS1* can also discriminate normal from tumor samples showing its diagnostic potential. Similarly, miR-141 expression can also robustly distinguish between cancerous from non-cancerous samples and hence has potential to be a diagnostic biomarker for RCC. Collectively, these results highlight the biomarker potential of *CDKN2B-AS1*/miR-141 expression in RCC, although it needs to be confirmed in a larger independent sample cohort.

Interestingly, we found that like lncRNA *CDKN2B-AS1*, cyclin-D1/D2 are also direct targets of miR-141. Accruing data proposes a new role for lncRNAs by pairing and sequestering specific miRNAs to protect repression of target mRNAs^32–35^. Our *in-silico* analysis showing *CDKN2B-AS1* shares regulatory miR-141 binding sites with cyclin-D1–D2 which was confirmed by luciferase and RIP assay. Cyclin-D1/D2 are also oncogenic in most cancers including RCC and play role in progression and metastasis^15,48,49^. Apart from the cell cycle regulation role of cyclin-D, it is also important in maintaining cellular adhesion, migration and metastasis through phosphorylation of paxillin and activation of Rac1^14,15,17^. Our results demonstrated that ectopic expression of miR-141 suppressed *CDKN2B-AS1* and cyclin-D1/D2 expression in RCC cell lines. We also observed a decrease in phospho-paxillin and Rac1 in miR-141 overexpressing cells compared to controls. Elevated paxillin phosphorylation is common in cancer tissues and is associated with tumorigenesis, EMT, and metastasis^15,16,50^. Paxillin interacts with numerous molecules, controlling the Rho family of GTPases, that are crucial regulators of adhesion dynamics^16,51^. Rac1 GTPase is required for cell migration and its hyperactivation results in cancer invasiveness and progression^52^. Similarly, we also found that knockdown of *CDKN2B-AS1* reduced cyclin-D1/D2 expression, thereby decreasing phosphorylation of paxillin and activation of rac1 to inhibit migration/invasion. Thus, *CDKN2B-AS1* functions as a ceRNA for miR-141 promoting cyclin-D1/D2 expression, resulting in RCC aggressiveness.

In summary, our study documents novel interactions between *CDKN2B-AS1*/miR-141 and cyclin D1–D2 in RCC. In view of our present in vitro and in vivo data, *CDKN2B-AS1*, cyclin-D1, and cyclin-D2 are shown to be direct and functional targets of miR-141 contributing to EMT by regulating rac1 and phospho-paxillin expression. Collectively, *CDKN2B-AS1*/miR-141 is identified as a novel interaction regulating tumorigenic progression and metastasis in RCC through the cyclin-D/Rac/paxillin network, and thus can be an attractive target for therapeutic intervention of aggressive RCC.

## Supplementary information

Supplementary Figure 1

Supplementary Figure 2

Supplementary Figure 3

Supplementary Figure 4

Supplementary Figure 5

Supplementary Figure 6

Supplementary Figure 7

Supplementary Figure 8

Supplementary Table 1

Supplementary Table 2

Supplementary Table 3

Supplementary figures and tables legends
