# Supplementary figures and images for: LncRNA CDKN2B-AS1/miR-141/cyclin D network regulates tumor progression and metastasis of renal cell carcinoma
Source: Cell Death Dis. 2020 Aug 19;11(8):660. doi: 10.1038/s41419-020-02877-0 (PMC7438482; doi:10.1038/s41419-020-02877-0)

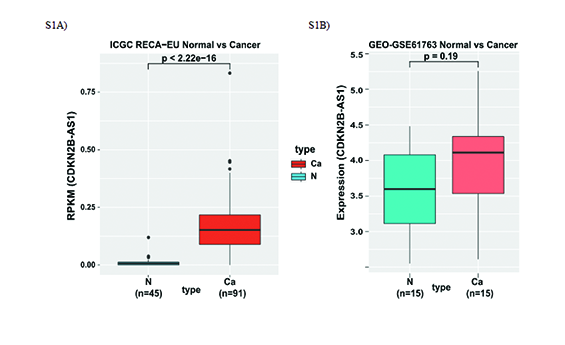

Supplement: Supplementary file 1 — Supplementary Figure 1 [file 41419_2020_2877_MOESM1_ESM.tif]

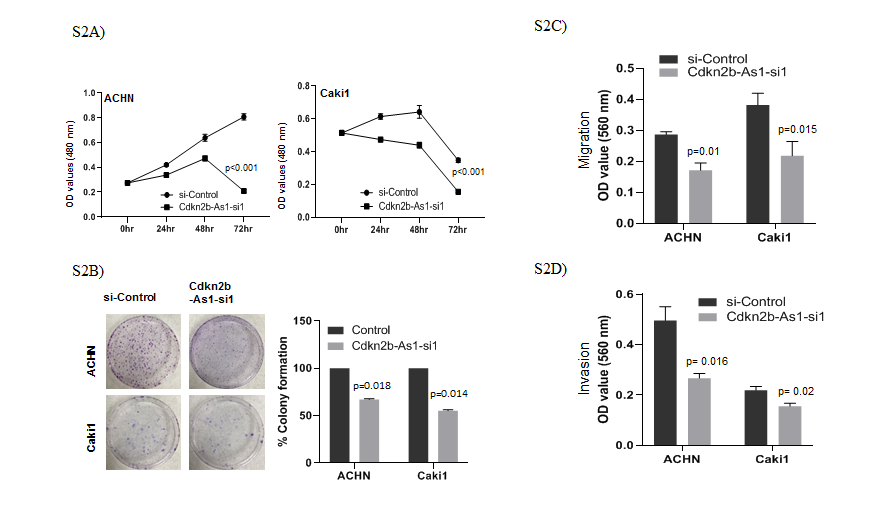

Supplement: Supplementary file 2 — Supplementary Figure 2 [file 41419_2020_2877_MOESM2_ESM.tif]

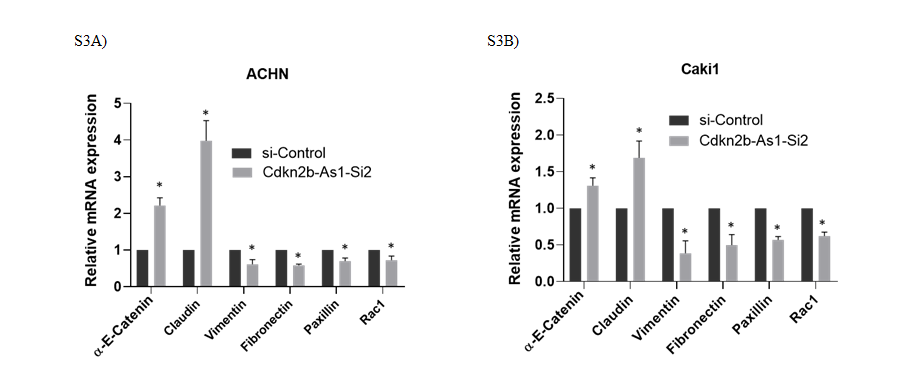

Supplement: Supplementary file 3 — Supplementary Figure 3 [file 41419_2020_2877_MOESM3_ESM.tif]

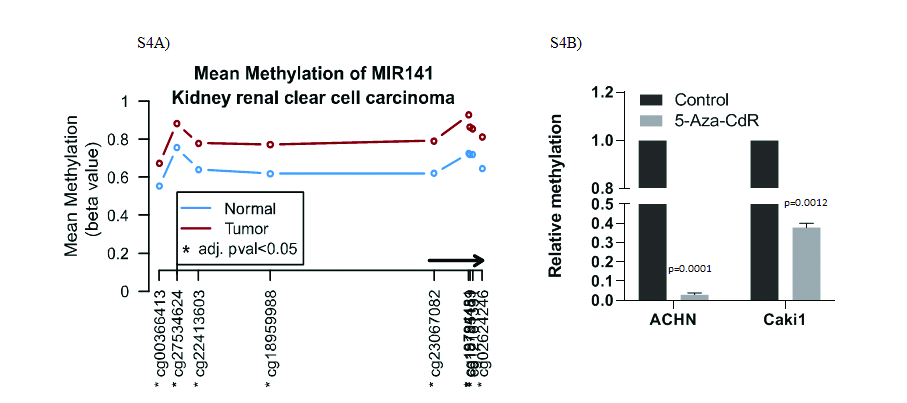

Supplement: Supplementary file 4 — Supplementary Figure 4 [file 41419_2020_2877_MOESM4_ESM.tif]

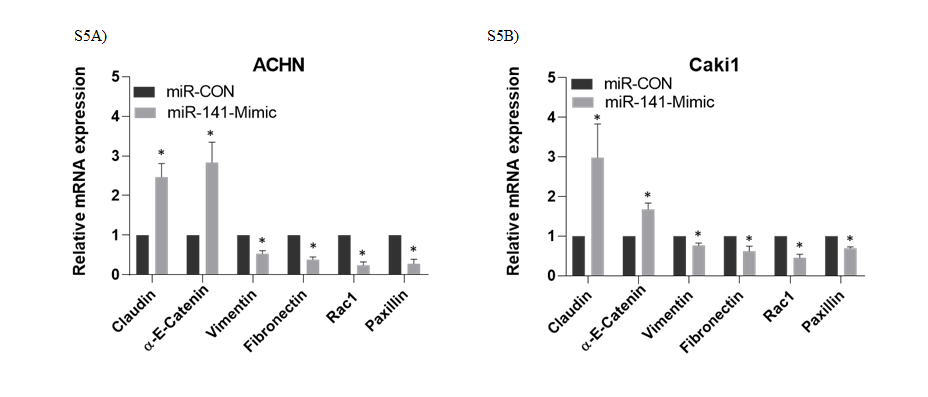

Supplement: Supplementary file 5 — Supplementary Figure 5 [file 41419_2020_2877_MOESM5_ESM.tif]

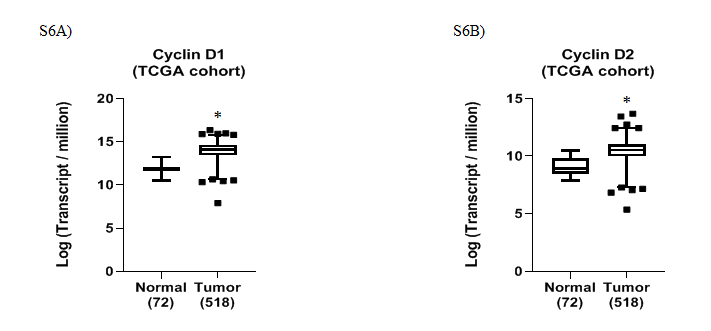

Supplement: Supplementary file 6 — Supplementary Figure 6 [file 41419_2020_2877_MOESM6_ESM.tif]

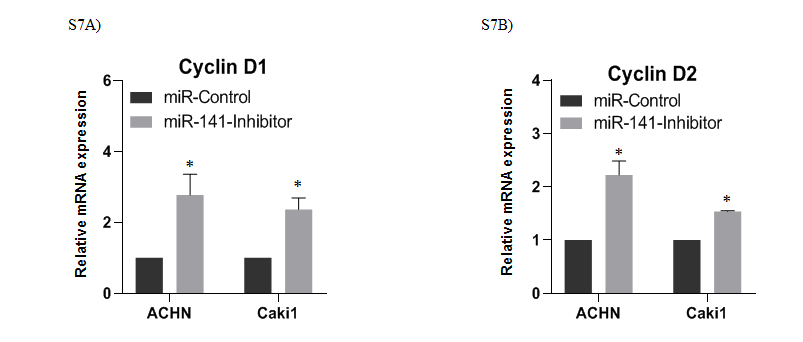

Supplement: Supplementary file 7 — Supplementary Figure 7 [file 41419_2020_2877_MOESM7_ESM.tif]

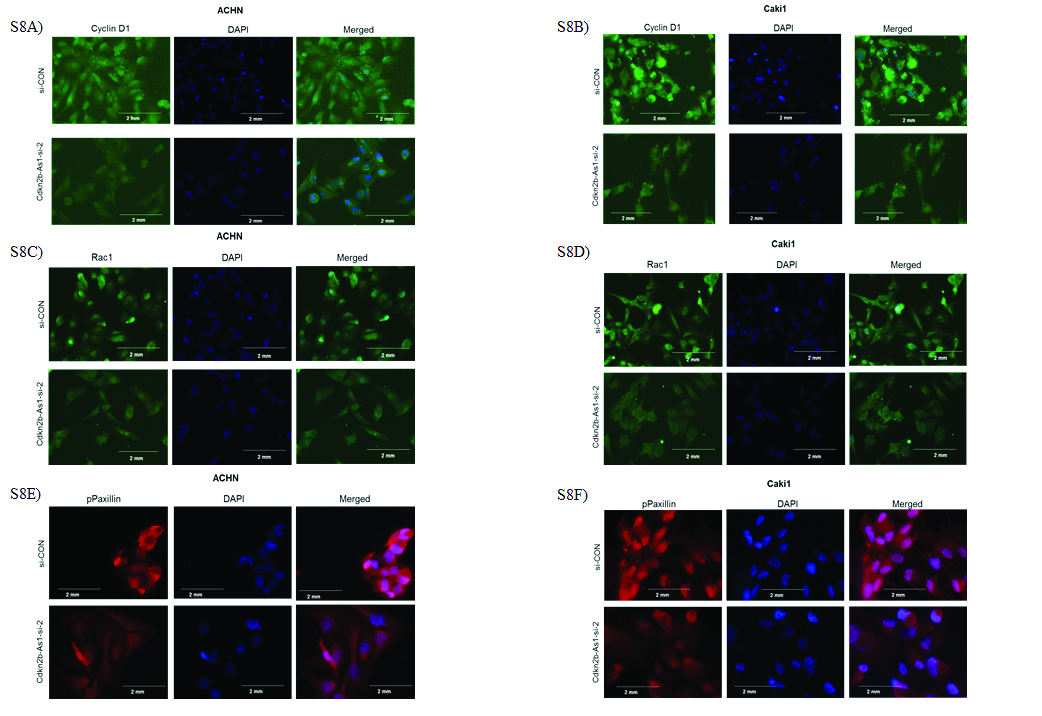

Supplement: Supplementary file 8 — Supplementary Figure 8 [file 41419_2020_2877_MOESM8_ESM.tif]
