# Supplementary material for: LncRNA CDKN2B-AS1/miR-141/cyclin D network regulates tumor progression and metastasis of renal cell carcinoma
Source: Cell Death Dis. 2020 Aug 19;11(8):660. doi: 10.1038/s41419-020-02877-0 (PMC7438482; doi:10.1038/s41419-020-02877-0)
Supplement: Supplementary file 9 — Supplementary Table 1 [file 41419_2020_2877_MOESM9_ESM.docx]

**Supplementary Table (T1)**

| **Primers Name** | **Sequences (3’🡪 5’)** |
| --- | --- |
| GAPDH-Forward | TCTTCACCACCATGGAGAAG |
| GAPDH-Reverse | GCAGAGATGATGACCCTTTTG |
| CDKN2B-AS1-Forward | AGGAGGCTGAATGTCAGTTTT |
| CDKN2B-AS1-Reverse | AGCGGTTTAGTTTAATTTCGCTT |
| α-E-Catenin – Forward | CCTCTGGAATTTAGCGCTCG |
| α-E-Catenin – Reverse | TCTCAACTGCCAGAGTCCTG |
| Fibronectin- Forward | ACAAGCATGTCTCTCTGCCAA |
| Fibronectin- Reverse | GCAATGTGCAGCCCTCATTT |
| Paxillin- Forward | AAAGTTGCGGGGCATAGACG |
| Paxillin- Reverse | AAGAACACAGGCCGTTTGGA |
| Rac1- Forward | AACCGGTGAATCTGGGCTTA |
| Rac1- Reverse | ATGCAGGACTCACAAGGGAA |
| Cyclin D1-Forward | TGGAGCCCGTGAAAAAGAGC |
| Cyclin D1-Reverse | TCTCCTTCATCTTAGAGGCCAC |
| Cyclin D2-Forward | ACTTGTGATGCCCTGACTG |
| Cyclin D2-Reverse | ACTTGGATCCGTCACGTTG |
| miR141-Meth-Forward | ATCGGTGTGTGTCGCGGGTC |
| miR141-Meth-Reverse | CTCGACCGTCGACCCGCCG |
| miR141-UnMeth-Forward | ATTGGTGTGTGTTGTGGGTT |
| miR141-UnMeth-Reverse | CTCAACCATCAACCCACCA |
