# Supplementary material for: LncRNA CDKN2B-AS1/miR-141/cyclin D network regulates tumor progression and metastasis of renal cell carcinoma
Source: Cell Death Dis. 2020 Aug 19;11(8):660. doi: 10.1038/s41419-020-02877-0 (PMC7438482; doi:10.1038/s41419-020-02877-0)
Supplement: Supplementary file 10 — Supplementary Table 2 [file 41419_2020_2877_MOESM10_ESM.docx]

**Supplemetary Table (T2)**

| **Primary antibodies** | **Company** | **Catalogue number** |
| --- | --- | --- |
| Cleaved-PARP | Cell Signaling | #5625 |
| Caspase3 | Cell Signaling | #9662, #9664 |
| Bax | Cell Signaling | #5023 |
| Bcl2 | Cell Signaling | #2876 |
| Cyclin D1 | Cell Signaling | #2978 |
| Cyclin D2 | Santa Cruz | Sc-593 |
| α-E-Catenin | Cell Signaling | #36611 |
| Claudin | Thermo Fisher Scientific | 37-4900 |
| Vimentin | Cell Signaling | #3390 |
| Fibronectin | Santa Cruz | sc-271098 |
| Rac | Cell Signaling | #2465 |
| Phosphor-Paxillin | Cell Signaling | #2541 |
| β-actin | Cell Signaling | #3700, #4790 |
| GAPDH | Santa Cruz | sc-32233 |

**Antibodies used:**
