# Supplementary material for: LncRNA CDKN2B-AS1/miR-141/cyclin D network regulates tumor progression and metastasis of renal cell carcinoma
Source: Cell Death Dis. 2020 Aug 19;11(8):660. doi: 10.1038/s41419-020-02877-0 (PMC7438482; doi:10.1038/s41419-020-02877-0)
Supplement: Supplementary file 11 — Supplementary Table 3 [file 41419_2020_2877_MOESM11_ESM.docx]

**Supplementary Table (T3)**

C**linicopathological characteristics of SFVAMC patient cohort (n = *68)**

**Clinicopathological No of cases**

**Feature**

**Age (years)**

 65 19

 ≥65 48

**Pathological T**

pT1 47

pT2 6

pT3 14

**Fuhrman Grade**

  II 43

III 20

IV 4

**Survival**

 Alive 50

Dead 17

* Clinicopathological characteristics of one patient was unavailable.
