# Supplementary material for: LncRNA CDKN2B-AS1/miR-141/cyclin D network regulates tumor progression and metastasis of renal cell carcinoma
Source: Cell Death Dis. 2020 Aug 19;11(8):660. doi: 10.1038/s41419-020-02877-0 (PMC7438482; doi:10.1038/s41419-020-02877-0)
Supplement: Supplementary file 12 — Supplementary figures and tables legends [file 41419_2020_2877_MOESM12_ESM.docx]

**Supplementary Figure Legends**

**Figure S1:** CDKN2B-AS1 expression levels of RCC patients in (A) ICGC and (B) GEO data cohort.

**Figure S2:** (A) Proliferation assessed by MTS assay after knockdown of CDKN2B-AS1 in ACHN and Caki1 cell lines with siRNA-1. (B) Knockdown of CDKN2B-AS1 with siRNA-1 significantly decreased colony formation in ACHN and Caki1 cells. (C-D) Reduced migration and invasion in CDKN2B-AS1 siRNA-1 transfected cells compared to controls.

**Figure S3:** Expression of α-E-Catenin, claudin, vimentin, fibronectin, paxillin and rac1 mRNA in ACHN (A) and Caki1 (B) cells after knockdown of CDKN2B-AS1 (*p <0.05).

**Figure S4:** (A) Average methylation for KIRC patient samples in TCGA cohort for all different probes (from TCGA wanderer). (B) Relative methylation in 5-Aza-CdR treated and untreated ACHN and Caki1 cell lines as assessed by MS-qPCR.

**Figure S5:** Expression of claudin, α-E-Catenin, vimentin, fibronectin, rac1 and paxillin mRNA in both ACHN (A) and Caki1 (B) cells overexpressing miR-141 (*p <0.05).

**Figure S6:** Expression levels of cyclin D1 (A) and cyclin D2 (B) of RCC patients in KIRC-TCGA data cohort (*p <0.05).

**Figure S7:** Expression levels of cyclin D1 (A) and cyclin D2 (B) mRNA in both ACHN and Caki1 cell lines treated with miR-141 inhibitor (50nmol/L) for 72 hours (*p <0.05).

**Figure S8**: Immunostaining of cyclin-D1 (A-B), rac1 (C-D) and pPaxillin (E-F) in ACHN and Caki1 cells after transfecting with si- CON/CDKN2B-AS1-si2. Left channel: target proteins (red/green), middle channel: DAPI (blue), right channel: merged, scale bar: 2mm.

**Supplementary Table (T1):** Forward and reverse primer sequences of genes used in this study.

**Supplementary Table (T2):** Details of the primary antibodies (company name and catalogue number) used in this study.

**Supplementary Table (T3):** Clinicopathological characteristics of SFVAMC patient cohort.
